# Atypical Spindle Cell/Pleomorphic Lipomatous Tumor with Atypical Imaging Features

**DOI:** 10.3390/diagnostics16132028

**Published:** 2026-06-29

**Authors:** Jiro Ichikawa, Tomonori Kawasaki, Kojiro Onohara, Masanori Wako, Rikito Tatsuno, Taro Fujimaki, Kouhei Mitsui, Tetsuhiro Hagino, Hirotaka Haro

**Affiliations:** 1 Department of Orthopaedic Surgery, Interdisciplinary Graduate School of Medicine, University of Yamanashi, Chuo 409-3898, Yamanashi, Japan; wako@yamanashi.ac.jp (M.W.); rtatsuno@yamanashi.ac.jp (R.T.); tfujimaki@yamanashi.ac.jp (T.F.); koumitsui@yamanashi.ac.jp (K.M.); t-hagino@yamanashi.ac.jp (T.H.); haro@yamanashi.ac.jp (H.H.); 2 Department of Pathology, Saitama Medical University International Medical Center, Hidaka 350-1298, Saitama, Japan; tomo.kawasaki.14@gmail.com; 3 Department of Diagnostic Radiology, Interdisciplinary Graduate School of Medicine, University of Yamanashi, Chuo 409-3898, Yamanashi, Japan; konohara@yamanashi.ac.jp

**Keywords:** atypical spindle cell/pleomorphic lipomatous tumor, histopathology, imaging, differential diagnosis

## Abstract

A 54-year-old female patient presented with a gradually enlarging mass on the ulnar side of the left wrist. Magnetic resonance imaging demonstrated a fat-containing lesion deep within the flexor carpi ulnaris that showed heterogeneous signal intensity, as well as weak internal and peripheral enhancement, which are not typical for atypical spindle cell/pleomorphic lipomatous tumors (ASPLTs). The imaging appearance overlapped with spindle cell lipoma (SCL), atypical lipomatous tumor/well-differentiated liposarcoma (ALT/WDL), and hibernoma, which made preoperative diagnosis challenging. The mass was excised en bloc. Histologically, the tumor consisted of mature adipocytes with substantial size variation, mild atypical spindle cells within a myxoid stroma, and scattered lipoblasts. Immunohistochemistry showed CD34 positivity and loss of RB1, while MDM2 amplification was absent on fluorescence in situ hybridization analysis, supporting a diagnosis of ASPLT. ASPLT is known for its broad range of morphological and radiological presentations, and this case further highlights the difficulty inherent in distinguishing it from SCL and ALT/WDL based on imaging alone. Recognition of its diverse features and the use of molecular testing are essential for accurately diagnosing ASPLT. Surgical excision remains the standard treatment. Although recurrence has been reported, metastasis of ASPLT is exceedingly rare.

**Figure 1:**
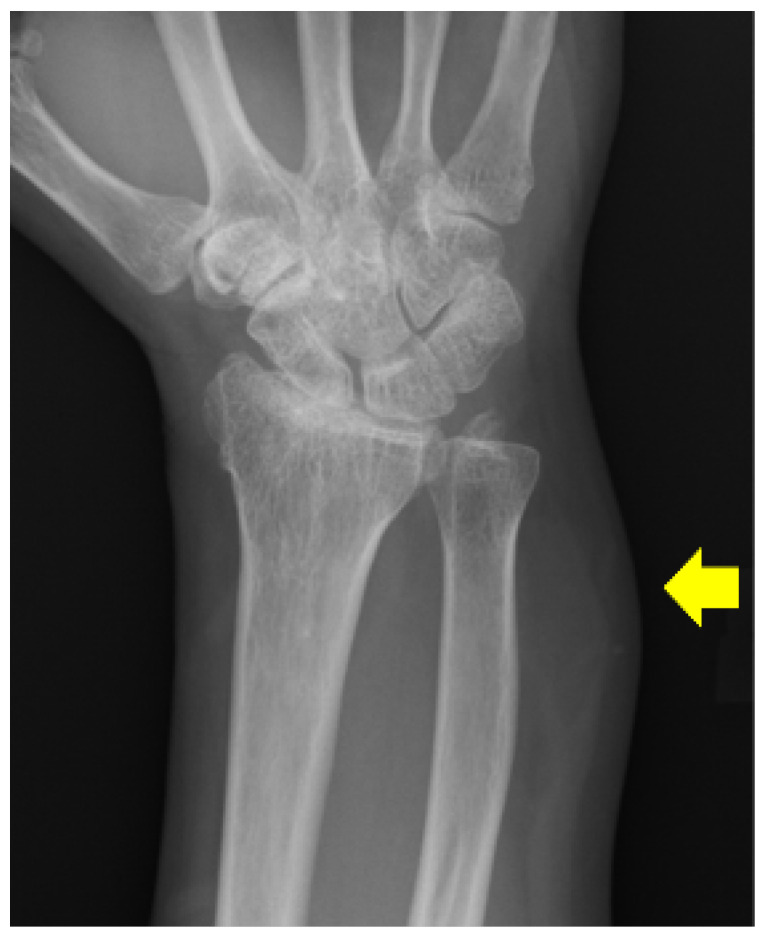
The patient was a 54-year-old woman who noticed a mass on the ulnar side of her left wrist, proximal to the ulnar head, ~6 months before her initial visit to our hospital. This mass gradually increased in size, prompting her to consult a local clinic, where she was referred to our institution for further evaluation. There was no redness or swelling of the overlying skin, and neither pain nor tenderness was present. Tinel’s sign was negative, and no limitation of the normal range of motion was observed. The patient had a history of two distal radius fractures of the left wrist caused by falls that had occurred four and three years prior. The first injury had been treated surgically, whereas the second was managed conservatively. On initial radiographs, a fat-dense lesion was observed on the lateral side of the ulna (yellow arrow), without evidence of calcification or bony deformity. Mild narrowing of the wrist joint space was noted, attributable to the previous distal radius fractures.

**Figure 2:**
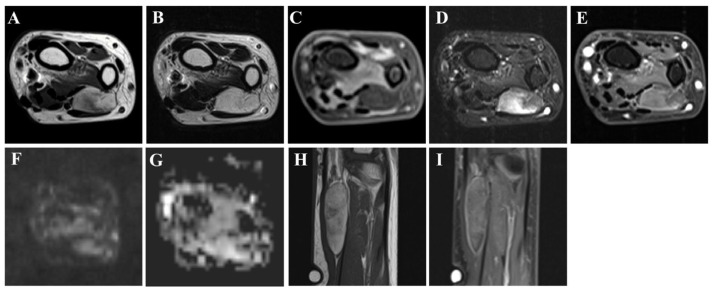
On magnetic resonance imaging (MRI), the lesion measured 35 × 20 × 12 mm and was located in the intermuscular space deep to the flexor carpi ulnaris. It contained macroscopic fat that was suppressed on fat-suppressed sequences, whereas its non-fatty components showed isointensity to muscle on axial (**A**) and sagittal (**H**) T1-weighted (3T; TR/TE = 600/13) and axial fat-suppressed T1-weighted images (**C**), as well as moderately high to high levels of signal intensity on axial T2-weighted (TR/TE = 4000/99) (**B**) and axial fat-suppressed T2-weighted images (**D**). Diffusion-weighted imaging (b = 1000) revealed mildly high signal intensity (**F**). The apparent diffusion coefficient within the lesion region of interest was 1.44711 × 10^−3^ mm^2^/s (**G**). The non-fatty components exhibited mild enhancement (**E**) axial and (**I**) sagittal after contrast administration, suggesting a fat-containing tumor with non-fatty components. The presence of non-fatty areas and mild enhancement made a simple lipoma unlikely. The lack of internal septa, small size, and high fat content made spindle cell lipoma (SCL) and hibernoma more likely than atypical spindle cell/pleomorphic lipomatous tumor (ASPLT) or atypical lipomatous tumor/well-differentiated liposarcoma (ALT/WDL) based on imaging findings. A preoperative biopsy was considered; however, due to the small size of the lesion, its deep location adjacent to the ulnar neurovascular bundle, and the potential risk of sampling error in a heterogeneous fat-containing tumor, complete excision was selected for both diagnostic and therapeutic purposes. Unlike in our case, even when malignancy is suspected, biopsy may be technically impossible in very small lesions. In such situations, even if the final diagnosis turns out to be a sarcoma, additional wide resection can be performed after the initial excisional biopsy [1]. A longitudinal incision was made along the ulnar aspect of the wrist, and the mass was identified deep to the flexor carpi ulnaris. Although the ulnar artery and ulnar nerve were in close proximity, they were easily preserved, and the tumor was removed en bloc.

**Figure 3:**
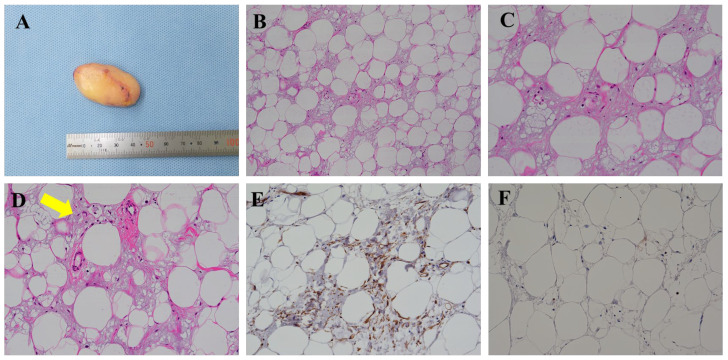
Macroscopically, the tumor was well-circumscribed, ovoid in shape, and yellow in color (**A**). Histologically, it consisted of mature adipocytes with substantial variations in cell size, accompanied by short spindle cells (yellow arrow) proliferating within a myxoid stroma. These spindle cells exhibited mild nuclear atypia but no mitotic activity. Scattered atypical lipoblasts were also observed ((**B**–**D**) using hematoxylin and eosin staining; (**B**) 100× magnification; (**C**,**D**) 200× magnification). Immunohistochemically, the tumor cells were positive for CD34 ((**E**) 200× magnification) and showed loss of RB1 ((**F**) 200× magnification).

**Figure 4:**
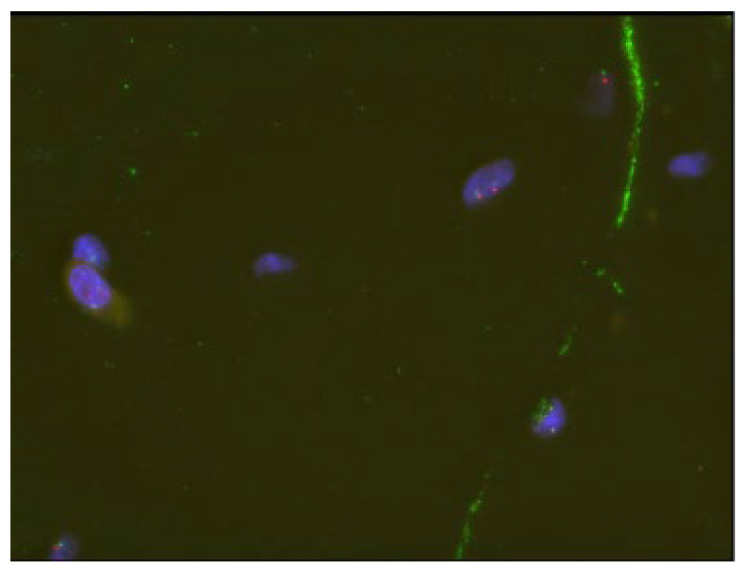
Fluorescence in situ hybridization (FISH) analysis for MDM2 (MDM2 (12q15) & Satellite Enumeration 12 Control Probe, Objective ×63) demonstrated no amplification (thirty cells were counted, with a MDM2/CEP12 ratio of <2 defined as negative in the FISH analysis). Based on these pathological findings, the tumor was diagnosed as an ASPLT. There were no postoperative symptoms such as numbness or limitations in the range of motion. At the 1-year postoperative follow-up, no evidence of disease recurrence was observed. Based on molecular pathological studies, ASPLT is recognized as: (1) a distinct entity separate from ALT/WDL and (2) a unified disease encompassing both atypical spindle cell lipomatous tumor and atypical pleomorphic cell tumor, according to the World Health Organization classification [2]. ASPLT is reported to arise with similar frequency in both subcutaneous and deep soft tissues [2,3]. Although ASPLT predominantly affects middle-aged men, the present case involved a 54-year-old woman, indicating that ASPLT can also occur in female patients. The tumor size (35 mm) was also smaller than the reported average (5–9 cm), further underscoring the variability in the presentation of ASPLT [3,4]. Typical MRI features of ASPLT include: (1) relatively low fat content (<25%), (2) conspicuous internal septations, and (3) variable but often appreciable enhancement [3]. In contrast, the present lesion demonstrated several imaging findings atypical for ASPLT. The lesion contained a relatively large amount of fat, lacked internal septations, and showed only mild enhancement. In contrast to the typical MRI phenotype of ASPLT, all three of these features favored a diagnosis of SCL rather than ASPLT, resulting in substantial overlap with other benign lipomatous tumors. Accordingly, SCL emerged as the principal radiological differential diagnosis [5]. Hibernoma was also considered because portions of the lesion demonstrated T2 hyperintensity and enhancement [6]. In addition, ALT/WDL could not be completely excluded because non-fatty components were present within the tumor [7]. Therefore, although MRI was useful for characterizing the lesion and narrowing the differential diagnosis, a definitive diagnosis could not be established based on imaging findings alone. Histopathological examination, together with molecular testing, was essential for establishing the diagnosis. The educational value of the present case lies in demonstrating that ASPLT may occasionally present with MRI findings more suggestive of SCL than those of conventional ASPLTs. Unlike conventional ASPLT, which frequently demonstrates relatively low fat content and internal septations, this lesion more closely resembled SCL and other benign lipomatous tumors. However, as suggested by the variability in its imaging features, the pathological features of ASPLT are also particularly diverse. ASPLTs are generally composed of atypical spindle cells, adipocytes, lipoblasts, and pleomorphic cells, embedded within a myxoid extracellular matrix with varying amounts of collagen [4]. The spindle cells typically show only focal and/or mild cytological and nuclear atypia. Lipoblasts are present in ~50% of cases, although typically in small numbers [4]. Immunohistochemically, CD34, S100, and desmin are frequently positive, with CD34 positivity reported in 60–70% of cases. Nuclear loss of Rb1 is observed in ~50–70% of ASPLT tumors [8]. MDM2 and CDK4 immunostaining may show weak and/or focal expression; however, amplification of MDM2 or CDK4 is typically absent, supporting distinction from ALT/WDL [2,4]. Although CDK4 amplification analysis was not performed, recent studies have shown that MDM2 amplification is more consistent and quantitatively higher than CDK4 in ALT/WDL, supporting the diagnostic reliability of MDM2 FISH alone [9]. To differentiate ASPLT from SCL, the absence or near-absence of atypical spindle cells and lipoblasts in SCL becomes an important diagnostic finding [8]. Conversely, because CD34 positivity and nuclear Rb1 loss are observed in nearly all SCL tumors, these markers are not practical discriminators between ASPLT and SCL [8]. Although surgical excision is generally recommended for ASPLT, selected cases involving small, indolent, and minimally symptomatic mesenchymal tumors may be managed with active surveillance to minimize treatment-related morbidity [10]. Local recurrence of ASPLT has been reported in approximately 10–15% of cases; however, metastasis has not been documented in the literature to date [2]. Although exceptionally rare, sarcomatous transformation has recently been described [11]. This case expands the recognized imaging spectrum of ASPLT and underscores a diagnostic pitfall when evaluating small fat-containing soft-tissue tumors.

## Data Availability

No new data were created or analyzed in this study.

## Abbreviations

The following abbreviations are used in this manuscript:

ASPLT	Atypical spindle cell/pleomorphic lipomatous tumor
SCL	Spindle cell lipoma
ALT/WDL	Typical lipomatous tumor/well-differentiated liposarcoma
MRI	Magnetic resonance imaging
FISH	Fluorescence in situ hybridization
